# Organizational Strategies for the Management of Intravenous Iron Therapy in Non-Hospitalized Settings: A Safe Opportunity to Implement Patient Blood Management in Italy

**DOI:** 10.3390/healthcare9091222

**Published:** 2021-09-16

**Authors:** Matteo Bolcato, Ivo Beverina, Daniele Rodriguez, Anna Aprile, Marco Trabucco Aurilio

**Affiliations:** 1 Legal Medicine, University of Padua, 35121 Padua, Italy; danielec.rodriguez@gmail.com (D.R.); anna.aprile@unipd.it (A.A.); 2 Blood Transfusion Center, Legnano General Hospital, ASST Ovest Milanese, 20025 Legnano, Italy; ivo.beverina@asst-ovestmi.it; 3 Department of Medicine and Health Sciences “V. Tiberio”, University of Molise, 86100 Campobasso, Italy; marco.trabuccoaurilio@unimol.it

**Keywords:** intravenous iron, anemia, iron deficiency, patient blood management, safety, clinical risk management, legal medicine

## Abstract

This article analyzes the recommendations issued by the Emilia Romagna region in July 2020 on “Organizational strategies for the safe management of intravenous iron therapy in patients in non-hospitalized settings”. The objective of these recommendations is to set up safe intravenous iron administration sites outside the hospital environment across the national territory. The document facilitates the organization of methods for intravenous iron infusion that are safe for the patient and correct from a medico-legal perspective. In addition, it opens the way for the widespread use of iron infusion in the field, providing benefits to patient quality of life. This program prevents unnecessary transfusions, reduces costs, prevents overcrowding in hospitals in the event of a pandemic, and enables patient treatment in the field, thus, saving on the use of personnel.

## 1. Introduction

Anemia is a global health problem especially in children of low-income areas, women of childbearing age, and the elderly. It is estimated that anemia affects more than two billion people worldwide, which accounts for more than one third of total world population [1,2].

Moreover, anemia (even mild–moderate) is independently associated with adverse patient outcomes and increases the risk of transfusions [3,4].

Red blood cell transfusion has been the default treatment for anemia. However, recent evidence demonstrates that transfusion may not be the most effective treatment for anemia and is itself an independent and additional risk factor for adverse patient outcomes [5,6].

In line with the main objective of improving patient outcomes, in recent years, the approach to treating anemic patients has changed significantly [7] with preference given to minimizing the use of blood products [8] by means of a vast range of proactive strategies [9].

In this context, the Patient Blood Management (PBM) program, defined as “an evidence-based bundle of care that optimizes medical and surgical patient outcomes by clinically managing and preserving a patient’s own blood” [10] has proven to have a significant impact on the need for blood transfusions, especially in patients with chronic conditions [11] undergoing elective surgical procedures. This innovation has created the necessity to change the methodological approach to patients and to organize activities in a more modern and up-to-date manner both in hospitals and in the field, thus, enabling personalized application of the three core pillars of PBM [12] for each patient. As a result, patient outcomes improve [13], the need for blood products decrease [14], and transfusion-related risks [15] and costs are minimized [16]. With regards to the last aspect, in the event of a shortage of financial resources, the correct usage of medical services can lead to benefits for health systems worldwide.

The first pillar concerns the optimization of red cell mass by stimulating erythropoiesis and treating modifiable underlying disorders [17].

The most common erythropoiesis disorder in the world population, affecting approximately 1.2 billion subjects including some in developed countries, is iron deficiency anemia (IDA) [1,2]. In addition to these figures is the number of people with isolated iron deficiency (ID without anemia) with those affected estimated to be double that of those with IDA [18]. Iron deficiency may cause serious anemia or less noticeable symptoms that can nevertheless cause disability and decreased working capacity and psycho-physical wellbeing. Conversely, the resolution of IDA-related conditions may increase clinical performance and reduce blood transfusions. In addition, it may serve as a protection against other diseases, particularly in women of childbearing age [19] and pregnant women, enabling them to sustain the fetus’ need for blood during pregnancy and protect it from deficiencies that can lead to retardation in the baby’s neurodevelopment [20,21].

The highest prevalence of anemia is reported in children within the first five years of life [22], in women of childbearing age [23], in patients hospitalized in Intensive Care Units [24], and in the elderly population due to other associated chronic conditions such as cancer, inflammatory bowel disease, kidney disease, and chronic heart failure [25,26,27]. Iron deficiency can further compromise their health condition and quality of life [27]. These facts clearly show the need to arrange public health programs and organizational models to create stable methods for administering iron in order to treat disease or deficiency-related conditions.

This is particularly important in Italy, since the National Institute of Statistics’ latest report shows that median survival will increase and by 2065, the average life expectancy could increase by more than five years for both sexes, reaching 86.1 and 90.2 years for men and women, respectively. This forecast shows a related change in the composition of the population by age: the aging population is projected to peak in Italy between 2045 and 2050, resulting in an over-65 population of approximately 34% and an average age of approximately 50. The demographic destiny for the rest of Europe and other developed countries appears to be similar, with an estimated over-65 population just short of 30% [28]. Therefore, the public health issue of IDA will become an ever-increasing factor in healthcare policies in European countries, especially considering the effect that an aging population has on blood availability due to an increase in the proportion of non-donating subjects [29]. For these reasons, it is imperative to devise suitable strategic solutions for IDA treatment.

Although oral iron therapy is considered the first line treatment, it takes time, and the different oral iron preparations vary in their tolerability and effectiveness. As many as 70% of patients who receive oral iron report significant gastrointestinal side effects. These symptoms may be severe enough to make the ingestion of oral iron therapy unpleasant or intolerable. In addition, some causes of iron deficiency will interfere with oral absorption of iron [30] as well as acute and chronic inflammation, which can lead to a huge decrease in iron bioavailability [26]. However, intravenous iron infusion has proven advantageous in facilitating rapid and sufficient iron supply and reintegrating physiological reserves [31,32].

In this regard, the guidelines issued by the National Blood Centre in Italy regarding the PBM [20] program recommend the use of single high-dose preparations for the repletion of iron in storage sites and that an evaluation of the patient’s blood tests should be carried out at least 30 days prior to the scheduled date for surgery. In these cases, infusions are usually performed in the hospital setting prior to hospitalization since some hospitals have designated outpatient clinics, provided by transfusion services, such as anemia clinics. These clinics may be appropriate and indeed advantageous in the hospital setting where anemia is treated in patients who will undergo major surgery shortly thereafter. However, access to such clinics may be limited for patients who need iron-infusion treatment for anemia but who have not been scheduled for surgery in the near future, especially in view of the current pandemic where access to hospital departments is limited for non-hospitalized patients.

## 2. Organizational Strategies for the Safe Management of Intravenous Iron Therapy in Patients in Non-Hospital Settings

The implementation of objectives set forth in the guidelines requires the creation of specific projects and documents. In Italy, the Health Service is organized into regions; although all regions are required to ensure the achievement of care levels set on a national level, each region has its own organizational and management autonomy in the healthcare setting, with the power to produce recommendation documents and enact laws with effect within the region’s jurisdiction.

The first region in Italy to produce such indications and recommendations on managing intravenous iron therapy in outpatient settings was Emilia Romagna. In July 2020, Guidelines for the Safety of Pharmacological Therapy were published on “Organizational strategies for the safe management of intravenous iron therapy in patients in non-hospital settings” [33]. The objective set forth in the document is to devise organizational strategies, protocols, and criteria for the management of intravenous iron therapy in patients treated in non-hospital care sites. It also considers the option of providing hospitals, research and care institutes, private accredited healthcare facilities, dialysis centers, community hospitals, care homes, nursing homes for the elderly, doctors of general medicine, and pediatricians in the field with indications regarding the opportunity to administer intravenous iron in non-hospital settings [34].

## 3. Recommendations

The introduction references the indications issued by the European Medicines Agency (EMA), which completed its review on the safety of using intravenous iron-containing medicines entitled: “New recommendations to manage risk of allergic reactions with intravenous iron-containing medicines” [35]. The products that were evaluated were ferric carboxymaltose, sodium ferric gluconate, and ferric saccharate.

The document concludes that overall, the benefits of these medicines outweigh the risks in treating iron deficiency where oral iron is not sufficient for or is not tolerated by the patient, provided that, during IV infusion, precautions are taken to minimize the risk of allergic reactions. 

By comparing the three iron-based medicines available in Italy, it is evident that these medicines present similar therapeutic indications but have different pharmacokinetic properties and a different iron content, which allows for the prescription of different dosage regimens. An example calculation is given for a person weighing 70 kg with Hb < 10 g/dl and, thus, a requirement of approximately 1.500 mg of iron. In order to reach the desired total amount with the maximum allowable dose per infusion, 12, 8, and 2 infusions are required for ferric gluconate, ferric saccharate, and ferric carboxymaltose, respectively. The frequency of administration has an impact on the organizational aspects and the probability of manifesting adverse reactions.

### 3.1. Patient Safety Recommendations

The safety recommendations regarding the administration of intravenous iron therapy are reported clearly and explicitly (Table 1). These recommendations are both useful and exhaustive and can be implemented swiftly in outpatient clinics and clinics outside the hospital setting, depending on the availability of qualified personnel such as doctors of general medicine and nurses trained to use the usual resuscitation cart equipment. No specific certificates or diplomas in specialized medicine are required, since the skills needed to recognize and treat potential adverse reactions are considered part of the cultural and practical expertise of every doctor and nurse.

### 3.2. Management Strategies

The document provides indications that all Local Health Units can use to devise safe management methods for use in non-hospital settings (Table 2).

### 3.3. Communication Recommendations

Communication between the various healthcare operators and between operators and patients is essential for guaranteeing the safety of and free consent to treatment. The creation of strategies on a local level, therefore, should include (a) defined communication methods between the various professionals involved in the various settings outlined in the organizational model along with definite operating procedures, and (b) activities to inform and involve patients, including information regarding potential adverse reactions.

### 3.4. Recommendations on Reporting Adverse Reactions and Events and Sentinel Events

Reporting adverse reactions (including staining or discoloration of the skin as a result of extravasation) to a drug alerts pharmacovigilance professionals to the potential risks related to that drug and allows regulatory bodies to intervene to ensure the safe and appropriate use thereof. Reports can be transmitted electronically via an online suspected adverse reaction report form available on the region’s dedicated website.

In the event of sentinel events, adverse events, and near miss events, healthcare personnel must follow the local organizational protocols for incident reporting in conjunction with the specific regional indications [36].

## 4. Conclusions

The recommendations issued by the Emilia Romagna region represent the first document in Italy to provide indications on the administration of intravenous iron. These indications highlight the considerable value of organizing methods for intravenous iron infusion that are safe for the patient and correct from a medico-legal perspective. Despite not containing all the potential solutions to this complex issue, it does provide useful indications for all facilities—local, regional, and national health units—that will need to adopt similar guidelines.

The recommendations pertaining to communication may form an integral part of a new pathway for information and consent to transfusions and of PBM strategies to avoid the administration of blood products as previously suggested [37].

In effect, it is the indications provided by international agencies, such as EMA, regarding patient safety [38] that are adopted. Such precautions are appropriate in line with the enactment of Law No. 24/2017 on the safety of care [39,40] in Italy, which focuses on activities for the prevention of adverse effects while treating patients. The same law recognizes safety of care as a fundamental right of all citizens [41].

In that context, the widespread use of PBM represents the search for a proactive approach to minimizing or eliminating transfusion-related risks [42]. The first pillar of PBM, as mentioned above, is the detection and management of anemia, which in the majority of cases is caused by iron deficiency. The objective, therefore, is to protect patients from the risks derived from the infusion of biological material. Though necessitating careful execution with a full understanding of the potential undesired effects [43,44], intravenous iron infusion is an excellent strategy for improving patient quality of life and reducing recourse to allogeneic transfusions. There are numerous articles [45,46,47,48] in support of the efficacy and safety of treating anemia with intravenous iron infusion. The document issued by Emilia Romagna opens the way for the extensive use of iron infusion in the field. The potential benefits include the opportunity to treat patients with IDA close to their homes, improve quality of life, avoid unnecessary blood transfusions, reduce, if not eliminate, transfusion-related risks, keep costs at a minimum [49,50], prevent overcrowding in hospitals during pandemics, and treat patients in the field, thus, saving on the use of personnel. Other countries such as Australia have already implemented a PBM program, which includes the administration of iron in outpatient settings by doctors of general medicine.

Whatever strategic organizational method is employed to create a network of safe iron infusion sites, transfusion medicine doctors remain the specialists of reference, playmakers in indicating and recommending the most appropriate therapeutic approach to treating anemic patients, which may increasingly lean towards infusion rather than transfusion.

## Figures and Tables

**Table 1:** Safety recommendations for intravenous iron therapy.

Monitor patients closely for signs and symptoms of hypersensitivity reactions during and following each infusion.
2. Drug administration should take place only when trained personnel are available to assess and manage anaphylactic reactions.
3. Ensure availability of equipment, drugs (including an injectable adrenaline solution 1:1000, antihistamines and/or corticosteroids), and medical devices to manage anaphylactic/anaphylactoid reactions as well as for cardiorespiratory resuscitation.
4. Keep the patient for observation for at least 30 min after each infusion.
5. Terminate treatment immediately in the event of hypersensitivity reactions or signs of intolerance.

**Table 2:** Synopsis of management strategies for intravenous iron infusion.

The prescriber must evaluate the case by discussing it with the medical transfusion services staff.
2. Each Local Health Unit must identify facilities where iron therapy administration can be organized and create a specific protocol indicating the equipment to be included in the emergency trolley.
3. First Aid training for facility personnel is mandatory.
4. Local Health Units may enter into collaboration agreements with other public or private bodies accredited with the necessary safety requirements in order to increase the number of suitable locations for administration.
5. Local Health Units will be able to evaluate the implementation of innovative organizational solutions such as providing a properly trained and equipped team to carry out administrations in remote locations or in facilities that do not meet the safety requirements set out in the company operating protocol (e.g., residential aged care facilities, patient homes).

## Data Availability

The datasets used during the current study are available from the corresponding author upon reasonable request.
